# Large variation in the movement of individual broiler chickens tracked in a commercial house using ultra-wideband backpacks

**DOI:** 10.1038/s41598-023-34149-0

**Published:** 2023-05-11

**Authors:** Mary Baxter, Niamh E. O’Connell

**Affiliations:** Institute for Global Food Security, School of Biological Sciences, Queens University Belfast, 19 Chlorine Gardens, Belfast, BT9 5DL United Kingdom

**Keywords:** Behavioural methods, Biological techniques, Physiology

## Abstract

Our understanding of the movement patterns of individual broiler chickens in large flocks is extremely limited. Here we report the use of a Real Time Locating System to track individual broilers in a house of 28 000 birds. Broilers were fitted with backpacks containing ultra-wideband tags on day 21 (N = 8 broilers) or day 24 (N = 9 broilers), with tags recording positioning and distance data until Day 38. Tagged birds were penned overnight on Day 31 to avoid ‘thinning’. We found no clear evidence of broilers consistently creating similar sized “home ranges”. Some broilers spent most time < 10 m from where they were originally found while others visited at least 90% of the house in the period before thinning. While some broilers rapidly returned to the area they were collected from at thinning, the majority did not. Movement data suggested that broilers that restricted themselves to smaller areas of the house were not necessarily less active. Although there was an average reduction in movement with age, this was not linear and there was individual variation. There was also no clear association between movement patterns and broiler weight or gait score, suggesting a more complicated relationship between activity, ranging and some welfare measures.

## Introduction

Commercially reared broiler chickens are housed in large, open, homogenous environments in flocks of several thousand birds. Given the size of the flocks, broiler chicken welfare is almost exclusively considered at the group level. Very little is known about how much individual broilers travel around the space available, whether they demonstrate a preference for particular areas and whether this is influenced by their gender, age, environment or personality. The technology has not been previously available to continuously track small animals in commercial housing, and studies have relied on colouring birds^1^ or using numbered leg tags and finding individuals at set time periods^2^. These methods can return varied results depending on flock size, pen size and monitoring intervals^3–5^. Indeed, there has been a long standing debate on whether poultry create “territories” in a commercial house^6–9^ or whether they use the space randomly^2^. In addition to answering these fundamental questions, continuous monitoring of individual broilers would allow us to more directly investigate the established link between reduced broiler activity levels and their increasing age, gait score, growth rate and body weight (reviewed by^10,11^).

Ultra-wideband (UWB) is a commercially available method of precision tracking that has now been miniaturised to the extent that tags can be carried by broiler chickens without significant impact to their normal behaviours^12^. Using a Real Time Locating System (RTLS), the XY coordinates of a broiler inside a commercial house can be provided by the system to an accuracy of less than 30 cm by calculating the time taken for a signal to travel from a transmitter tag to several receivers or “anchors”. We recently validated this technology for use in broiler housing^12^ and have further developed an algorithm to trim the data and improve the quality of movement estimates. To the best of our knowledge, in this paper we describe the first use of this technology in a commercial indoor broiler house to continuously track individual birds. The main aims of this study were to (1) determine how far individual broilers move around a commercial house and whether they display preferences for certain areas of the house, suggesting a “home range”, and (2) to explore the association between movement and elements of their physiology, including body weight, gait score and age.

## Materials and methods

### Animals and housing

This study was conducted in November 2020 on a Moy Park affiliated “Higher Welfare” farm in Northern Ireland. Broilers reared on Higher Welfare farms are kept at a lower stocking density and provided with additional environmental enrichment compared to Standard farms. One commercial house, 85 m by 20 m, was stocked with 28,000 day old Ross 308 broilers at the beginning of the production cycle. The house was metal framed and fitted with windows along both long sides, providing natural daylight during daylight hours (approximately 9 00 h to 16 00 h at this time of year). The chicks were placed “as-hatched”, giving an approximate 50:50 ratio of male:female broilers. There was an approximate 1700 m^2^ of available floor space, with a stocking density that did not exceed 30 kg/m^2^. As was standard for this farm, straw bales (1.5 per 1000 birds) and platform perches (3 along one side of the house and 4 along the other) were provided as additional environmental enrichment. Platform perches were 260 cm by 60 cm and were raised at the farmer’s discretion as birds aged, to a max height of approximately 30 cm from floor level. The chopped straw bales were plastic wrapped, with the plastic cut open across the cycle to allow the birds access to the straw. Food and water were supplied ad libitum from rows of bell feeders and nipple drinkers, respectively. The house was bedded with straw crumb at the beginning of the cycle and additional woodshavings were distributed at the farmer’s discretion to maintain litter condition. As standard, broilers were partially depopulated (thinned) during week 5 (Day 32) and the remaining birds were cleared for slaughter in week 6 (Day 40) of the production cycle.

### Ultra-wideband system

**Figure 1:**
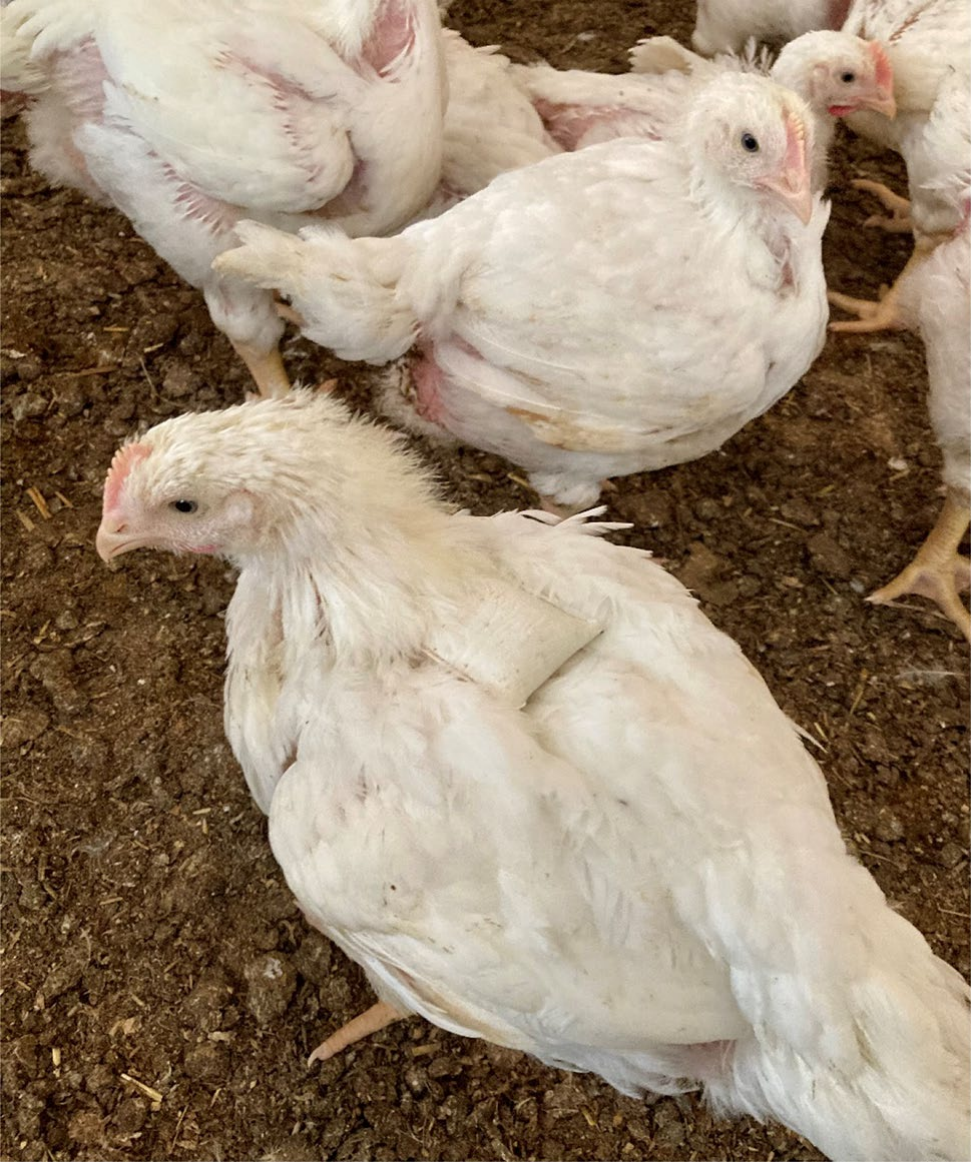
A Ross 308 broiler chicken wearing a backpack containing an ultra-wideband tag.

A commercially available RTLS based on UWB technology was fitted into the broiler house to allow for individual tagged broilers to be tracked in real-time. The system was manufactured by SEWIO (Brno, Czech Republic) and supplied by Locatify (Reykjavík, Iceland). It consisted of a network of twelve receivers or “anchors” and UWB transmitters or “tags”. The tags transmitted positioning data to the anchors, which were fitted to walls of the commercial house in three sets of four, virtually dividing the house into thirds^12^. Anchor data, tag visualisation and positioning data were provided through an application programming interface (API), RTLS Studio (SEWIO, Brno, Czech Republic). The Piccolino UWB tags used were 29 mm × 37 mm and weighed 14 g. They were placed into white, matt, PVC, water resistant “backpacks” (Fig. 1). The straps that went under the broiler’s wings were made of 3 mm braided polyester and fitted with an adjustable toggle to allow alterations as the birds grew. Together with the tags, the backpacks were 60 mm × 40 mm and weighed a total of 19 g, which was 1.4% of the weight of the average three-week old broiler (~ 1.4 kg). This is below the recommended limit of 5% additional weight added to animals during tracking studies^13^.

**Figure 2:**
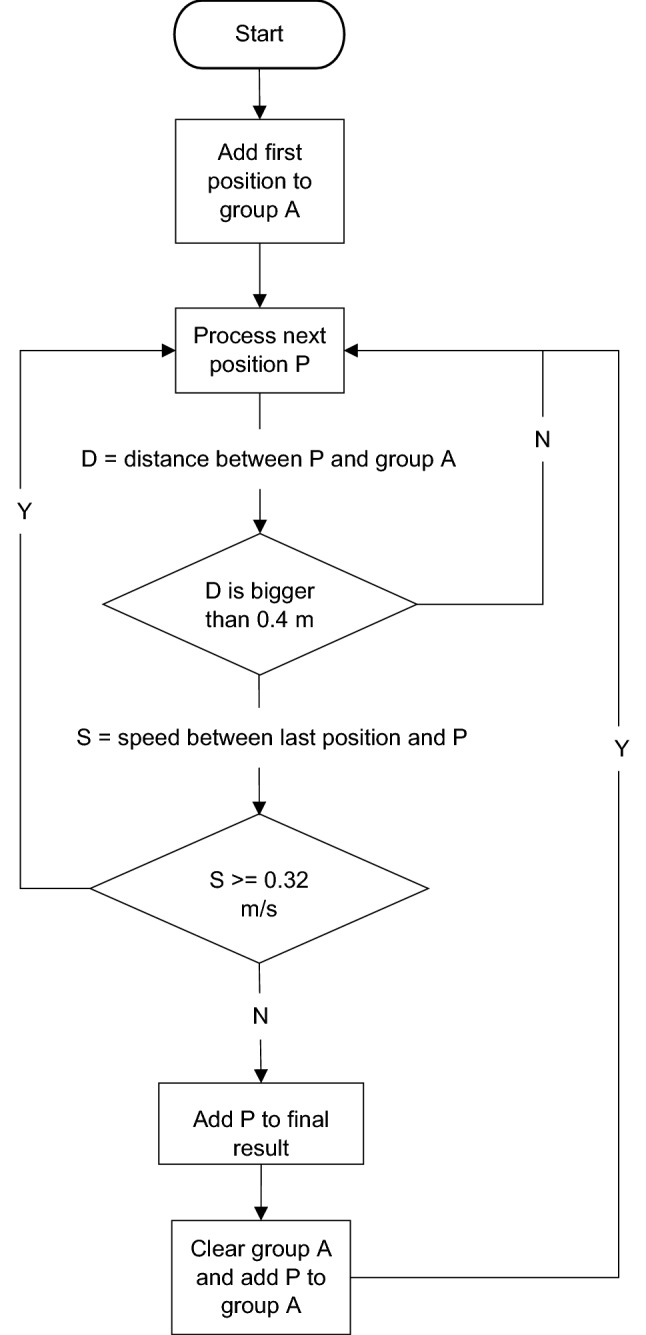
Representation of the algorithm used to filter positioning data from UWB tags fitted onto broiler chickens. The process involves adding the first position to Group A, then process the next position (P) and if the distance between Group A and P is smaller than 0.4 m (i.e. the distance moved between two positions is < 0.4 m) then P is added to Group A. If it is larger than 0.4 m then the speed between the last position and P is calculated, if this is more than 0.32 m/s then that position is discarded and the next position processed. This removes “unlikely” data errors (e.g. moving rapidly away and then back to the same position). If the speed is less than 0.32 m/s then P is added to the final data set and the process continued with the next data point.

The ability of the system to locate individual broilers moving freely in the house and to accurately track their movements was previously assessed by Baxter and O’Connell^12^. In brief, we found that broilers could be easily located among the flock and that the system was able to accurately report the tagged bird’s location. However, distances travelled tended to be exaggerated as a result of small deviations in location of  < 30 cm being recorded while the bird was relatively stationary. We also detected a number of false positions as a result of interference in the transmission of positioning data, e.g. the tag moved 20 m away and then back to its previous location rapidly. During testing, tags had been set to record positioning data 10 times per second. Despite some interruptions in data and periods of “sleep” when the broilers were immobile enough to trigger the tags to stop recording data, tags still generally recorded several positioning data points per second for the majority of the time they were fitted. This resulted in large amounts of data that inflated distance measures and contained “unlikely” movement patterns where positioning data was briefly inaccurate. Therefore, in collaboration with Locatify (Reykjavík, Iceland), an algorithm was developed to filter data, average positioning data per second and remove unlikely data points (Fig. 2). K-means algorithm was initially tested but was found to be ineffective. Over a period of several months, broilers were tagged and video recorded for short periods of time while the algorithm was refined. Movements were synchronised against data with various filtering parameters and alterations made to the algorithm as needed to most closely match the broiler’s movement.

### Tagged broilers

Broilers were fitted with backpacks in two stages to allow for initial monitoring of the system’s capacity, with backpacks fitted on either the morning of Day 21 or Day 24, and removed on Day 38. On Day 21 and again on Day 24, 12 broilers (24 in total) were selected from six randomly identified virtual zones of the house, balanced for central and edge zones (three of each). There were one hundred virtual zones, each measuring 4.26 × 4.02 m. Two broilers from each section with a gait score of 0 or 1^14^ were chosen and weighed by placing them in a container attached to a digital scale (Dr Meter Fishing Scale, Shenzhen Thousandshores Technology Co. Ltd, Guangdong, CN). They were then fitted with a backpack containing an UWB tag that was assigned a number from 2 to 25. The observer carried a tag (Tag 1) and tablet to visualise their location on the system. As expected, some broilers demonstrated initial abnormal movements, preened and/or remained immobile immediately after the backpack was fitted. Based on our initial study, broilers were expected to acclimatise quickly to the backpacks^12^, with any abnormal behaviours expected to have resolved 24–48 h after the backpack was fitted. All broilers were checked 24 h later, and then daily for the duration of the study. On each day, tagged broilers were located and monitored to detect any persistent abnormal behaviours or difficulty coping with the backpack. The fit of the backpack was checked and wingstraps loosened as needed. Due to their rapid growth, daily monitoring was essential to allow for backpacks to be adjusted as birds grew.

On the evening of Day 31, all tagged broilers were collected by the observer and penned in one corner of the house to prevent their removal during thinning. As the birds are collected during thinning in very low light and the backpacks were difficult to see, it was not possible to instruct staff to leave any tagged broilers they found. The pen included several feeder bulbs and a drinking line; all broilers were in visual contact with the rest of the flock at all times. Thinning occurred in the early hours of Day 32. At approximately 09 00 h, farm staff opened the pen and released all tagged broilers into the thinned flock. On Day 38, all tagged broilers were located, gait scored and weighed before backpacks were removed.

### Data collection

**Figure 3:**
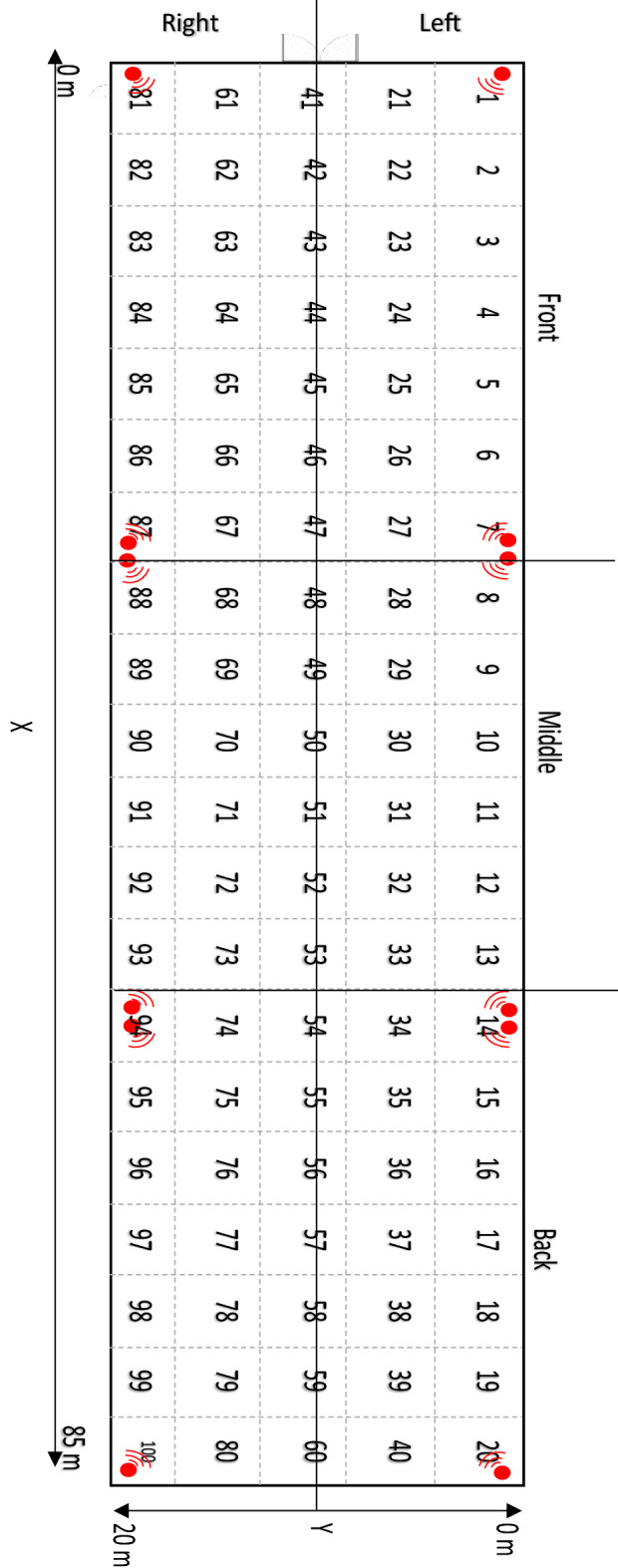
A schematic of the broiler house depicting the location of the anchors (red circles) and the virtual zones and areas used for analysis. The 100 small zones were 4.26 by 4.02 m. Zones 1–20, 40, 60, 80, 21, 41, 61 and 81–100 were considered to be edge zones, the remaining zones were central zones. The house was divided into thirds; front, middle and back. The left and right side of the house was divided down the central line from front to the back.

Data collection began 24 h after broilers had been tagged to allow for an acclimatisation period. For tags 2–13 this began at 12 00 h on Day 22 and for tags 14–25 this began at 12 00 h on Day 25. Filtered data was stored on a remote server for analysis. Diagrams, including heat maps and movement maps, were generated using the API (RTLS studio, SEWIO, Brno, Czech Republic). All further analysis was performed by exporting spreadsheets of data consisting of time-stamped XY coordinates of each tag. To get an overview of how the house was used and what percentage was covered, the house was virtually split into 100 small zones (4.26 m by 4.02 m) and three main areas (front, middle and back; each around 28 m by 20 m; Fig. 3). Zones were further classified as edge zones (one side of the zone touching the wall of the house) or central zones (all non-edge zones). The house was also divided virtually into a left and right side along the central line from the front to the back (Fig. 3).

Data from the pre-thinning period was used to determine the undisturbed movement patterns of broilers, consisting of positioning data from 12 00 h on Day 22 or Day 25 until 12 00 h on Day 31 (9 or 6 days of data). Analysis of movement post-thinning consisted of the period from 09 00 h on Day 32 until 09 00 h on Day 38 (6 days). Where analysis covered both periods for the total distance recorded, this excluded the period of time when broilers were penned for thinning. Where data is presented by day, only days where data was recorded from 00 00 h to 00 00 h are presented, i.e. excluding days where data collection began or ended at 12 00 h (Days 22, 25 and 31) or 09 00 h (Days 32 and 38).

### Statistical analysis

Results reported are largely descriptive. Unless stated, N refers to the number of tagged broilers. Pearson’s correlation coefficient was used to explore the level of association between the total pre-thin distance travelled by broilers and the number of small zones they were detected in (N = 17). For pre-thinning associations between physiology and movement, Pearson’s correlation was used to analyse data separately for tags 3–13 (N = 8) and 14–25 (N = 9), as these tags recorded data for differing lengths of time.

### Ethics declaration

The study was approved by the School of Biological Sciences (Queen’s University Belfast) Research Ethics Committee (reference number: QUB-BS-AREC-19-005) and performed in accordance with relevant guidelines and regulations. Reporting in the manuscript follows recommendations in ARRIVE guidelines.

## Results

The UWB system used in this study was an effective method of tracking and locating group housed broilers. All broilers could be identified and inspected daily, and almost all resumed normal behaviours quickly after being fitted with the tags. A total of seven out of the 24 backpacks were not able to provide data over the course of the study. Four backpacks fell off and were found on the floor of the house, and three backpacks were removed due to (1) a wingstrap causing rubbing, (2) the broiler demonstrating persistent crawling, and (3) the broiler not resuming normal behaviour beyond 24 h. No tagged broilers died or were culled during the study. Distances were calculated as the direct distance between one coordinate and the next in metres. We report these as “recorded distances” rather than true distances, as it was not possible to validate these distances against observed broiler movement over long periods of time and inflation is likely due to the accuracy level of the system. We previously found a positive correlation between true distance and recorded distance in data analysed without the algorithm in place^12^. Although the algorithm used in this study did remove the majority of unlikely data points, we cautiously consider them to be more useful in identifying overall trends rather that considering them to be exact distances travelled by the broilers.

### Broiler movement patterns

#### Use of the space available before thinning

The majority of tagged broilers were recorded in each of the front, middle and back of the house at some point during the pre-thinning period, with 59% of broilers recorded in these three main areas, 29% present in two areas and 12% remaining inside only one area. All tagged broilers were detected in both the left and right side of the house. A total of 44% of the broilers tagged for six days were detected in more than half of the house, with this increasing to 75% for those tagged for the longer nine day period (Table 1).

**Table 1:** The movement of Ross 308 broiler chickens wearing ultra-wideband tags in an indoor commercial broiler house (85 × 20 m) before partial depopulation (thinning).

Tag^1^	Weight		Gait score^2^		Location broilers were initially tagged^3^		Number of areas visited^4^	% of Small Zones visited^4,5^	% time spent in each area of the house^4^			% time spent in central or edge zones^4^		Recorded distance pre-thin (m)^4,6^	Total recorded distance (m)^6^
	Start (g)	Final (g)	Start	Final					Front of the house	Middle of the house	Back of the house	Edge	Central		
3	1050	3190	1	2	Front	Edge	2	28	99.9	0.1	0	57.7	42.3	11 118	17 638
5	1050	3240	0	2	Front	Edge	3	73	47.1	50.4	2.5	58.2	41.8	13 091	18 535
7	875	2990	0	2	Back	Central	3	82	37.6	22.8	39.6	42.3	57.7	11 760	18 668
8	1075	3380	1	2	Front	Central	3	76	33.3	53.4	13.3	15.9	84.1	12 423	17 370
9	890	3000	0	2	Front	Central	2	41	97.6	2.4	0	49.3	50.7	12 411	17 460
10	965	2920	0	1	Back	Edge	3	97	37.7	23.1	39.2	46.1	53.9	10 714	14 537
12	1105	3710	1	2	Back	Central	3	58	1.5	41.5	57.0	35.8	64.2	10 390	16 259
13	1180	3700	1	2	Back	Central	3	65	0.1	31.2	68.7	52.0	48.0	8 949	15 859
14	1280	3260	1	2	Front	Edge	2	66	0	48.0	52.0	54.9	45.1	6 484	12 481
15	1105	3020	0	2	Front	Edge	2	37	99.3	0.7	0	89.8	10.2	12 103	19 554
16	1370	3780	1	2	Front	Central	1	21	100	0	0	88.3	11.7	13 438	23 802
18	1175	3200	1	1	Back	Central	3	90	8.3	20.8	70.9	41.9	58.1	8 206	14 339
19	1230	3330	1	2	Back	Central	3	49	0.7	11.9	87.4	68.6	31.4	5 536	11 242
22	1190	2950	1	1	Front	Central	3	71	55.2	41.8	3.0	43.9	56.1	10 852	17 679
23	1400	3780	1	2	Front	Central	1	21	100	0	0	65.8	34.2	8 844	17 116
24	1190	3140	0	1	Back	Edge	2	43	0	28.2	71.8	71.0	29.0	6 186	14 379
25	1410	3710	1	3	Back	Edge	3	69	46.0	21.1	32.9	45.5	54.5	8 042	15 629
Av								58.1	45.0	23.4	31.7	54.5	45.5		

The tagging of birds was staggered, with tags 3–13 observed for nine days from Day 22 until Day 31 and tags 14–25 observed for six days from Day 25 until Day 31. Data collection continued after thinning and total recorded distance consists of both the pre-thinning period (9 or 6 days) and the post thinning period (7 days).

^1^A total of 24 broilers were initially tagged, full data sets available for 17.

^2^Gaits were scored according to the Welfare Quality 2009 protocol for broilers; 0—normal, dexterous and agile, 1—slight abnormality, but difficult to define, 2—definite and identifiable abnormality, 3—obvious abnormality, affects ability to move, 4—severe abnormality, only takes a few steps, 5—incapable of walking.

^3^Broilers were initially tagged in small zones (4.26 × 4.02 m) that were in one of three areas; front, middle or back (~ 28 × 20 m each). They were also either edge (at least one side of the zone consisted of the house wall) or central.

^4^Measurements taken before thinning.

^5^Small zones were 4.26 × 4.02 m.

^6^This is the distance recorded by the ultra-wideband system which has an accuracy of ~ 30 cm. As such, distances may be inflated and are used to represent patterns of movement rather than exact distances travelled.

**Figure 4:**
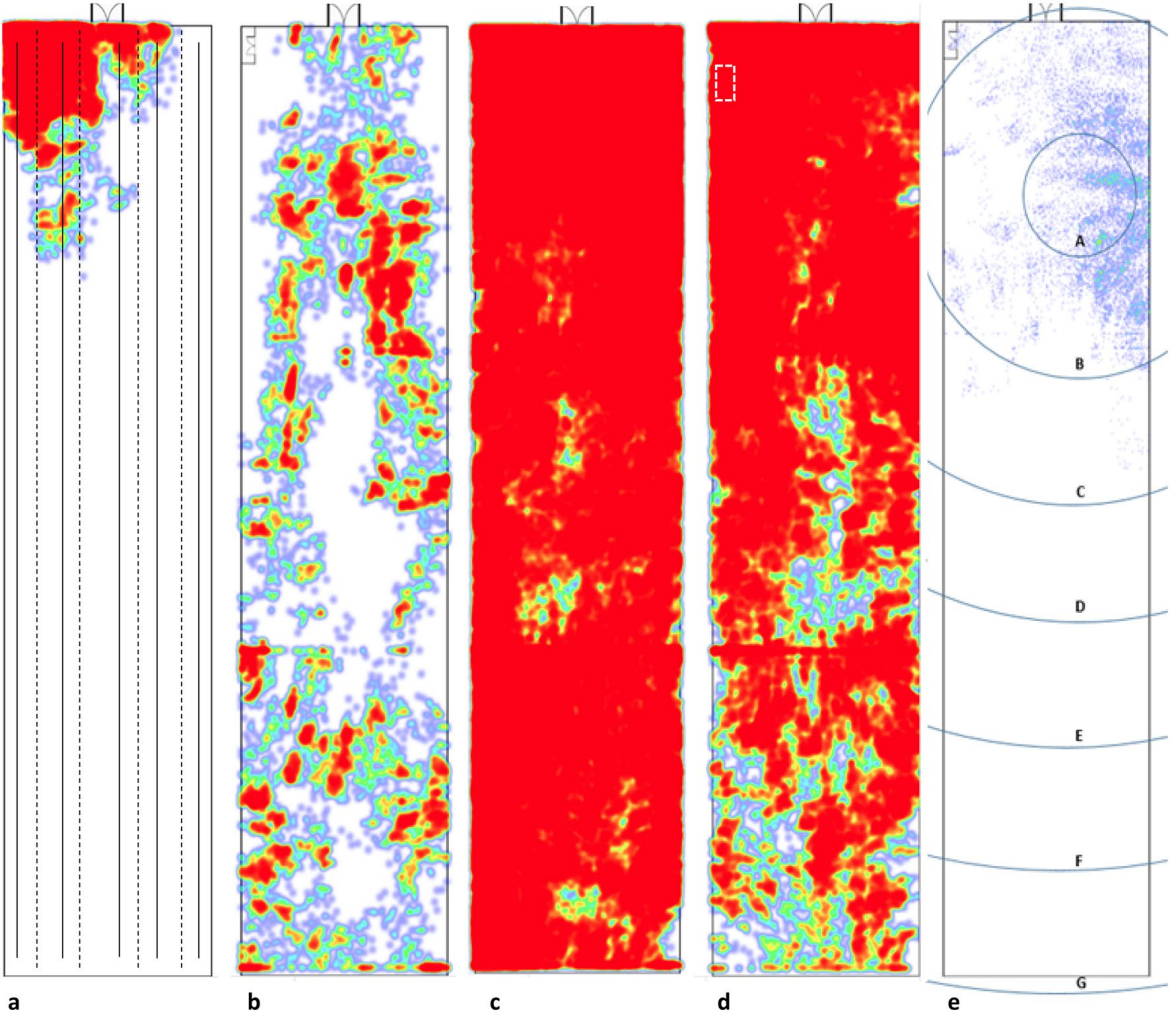
Diagrams (**a**–**d**) display heat maps created for (**a**) tag 16 (one of the lowest ranging broilers), (**b**) tag 10 (the highest ranging broiler), (**c**) all tags in the pre-thinning period and (**d**) all tags in the post-thinning period after they had been released from a pen in the top left hand corner of the house (white box). Darker (red) areas represent areas with a higher density of data points, which indicates that broilers spent more time in that area. Diagram (**a**) contains an indication of the approximate location of feeder and drinker lines; solid lines indicate drinkers and dashed lines indicate feeders. Diagram (**e**) represents an example of the inclusion areas used to determine how much time broilers spent in proximity to the centre of the zone they were initially tagged in. Data for Tag 9 are displayed in this diagram, with letters A–G representing 10 m to 70 m distance from the initial tagging zone, respectively.

During the pre-thinning period, the lowest ranging broilers were recorded in 21 zones and the highest ranging was recorded in 97 out of a possible 100 virtual zones, using 21% and 97% of the house when expressed as a percentage (Table 1; Fig. 4a,b). Overall, tagged broilers visited an average of 58% of the house (Table 1). Broilers tagged for six days were recorded in an average of 52 zones while those tagged for nine days before thinning were recorded in an average of 65 zones. Overall, broilers spent an average of 54.5% of their time in edge zones and 45.5% of their time in central zones (Table 1). This was despite there being more central zones (54) than edge zones (46). However, there was also significant individual variation in this measure, with the total time spent in edge areas varying from 16 to 90% (Table 1).

**Figure 5:**
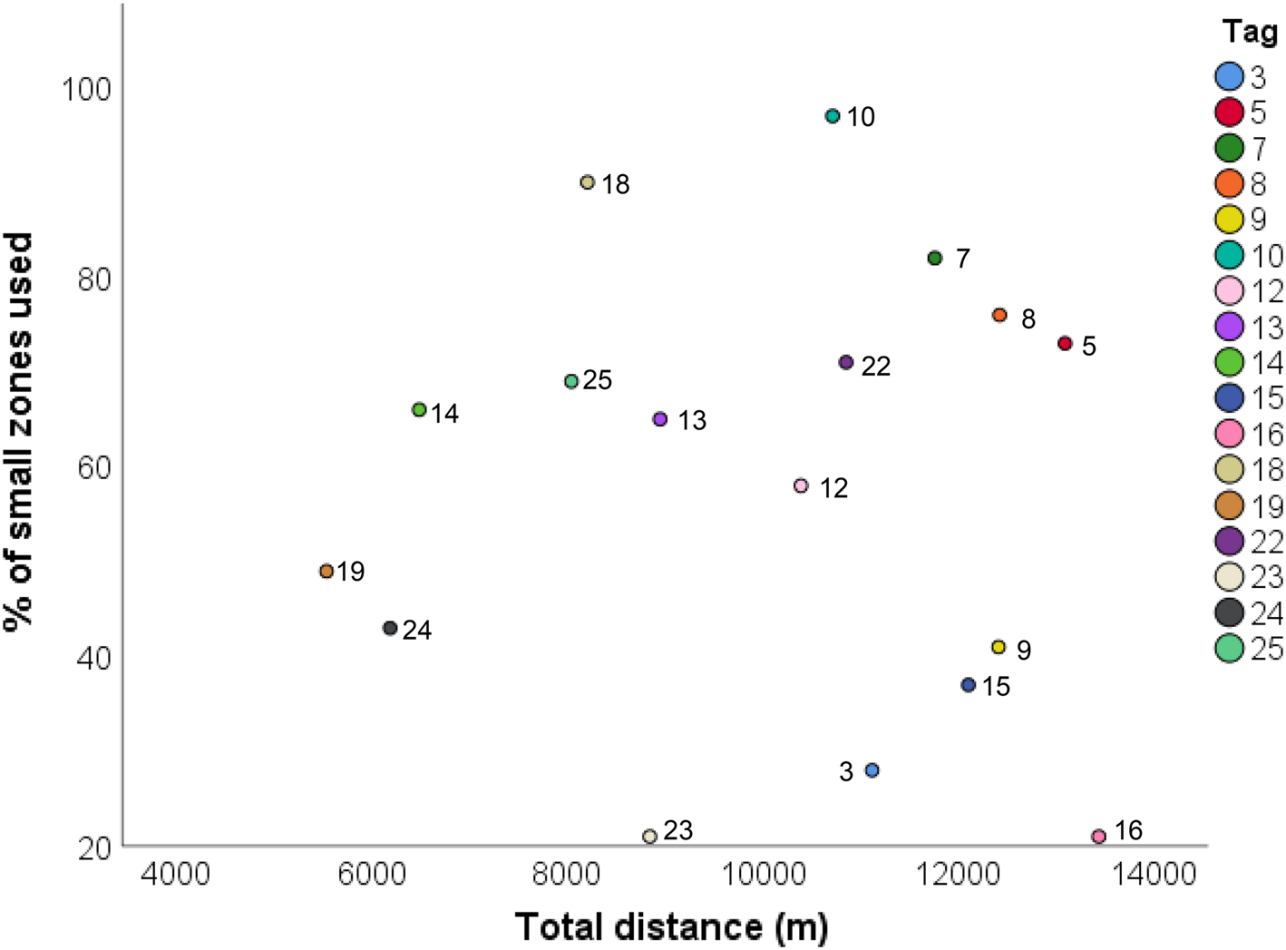
Scatter plot for the total distance broiler chickens were recorded moving against the percentage of the house they were detected in. Broilers could travel into a total of 100 virtual zones (4.26 × 4.02 m) across the observation period. For tags 3 – 13 this was a nine day period from Day 22 until Day 31 and for tags 14–25 this was a six day period from Day 25 until Day 31. Total distance recorded is likely to be inflated rather than an accurate representation, as the UWB system had a ± 30 cm accuracy.

Interestingly, there was no correlation between the total recorded distance and the number of zones that broilers were detected in (N = 17; p > 0.8; Fig. 5). The broiler that travelled the furthest distance (Tag 16) also occupied the smallest number of zones, suggesting that it restricted its movement to a smaller area but still maintained a relatively high level of activity. Conversely, the highest ranging broiler (Tag 10) detected in 97% of zones recorded a lower distance travelled than several broilers that occupied smaller areas. As the bird carrying Tag 16 was the only one to remain exclusively near the house entry door, it is possible that regular disturbance by farming staff and observers led to the distance recorded being inflated. However, with Tag 16 removed from analysis as an outlier, there remained no significant correlation between recorded distance and the number of zones covered (N = 16; p > 0.6).

#### Exploring the existence of location preference before thinning

There was some indication of a general location preference, with 65% of tagged birds spending more than half of their time in the same main area that they were initially tagged in (front, middle or back of the house; Table 1). Only 4 out of 17 of tagged broilers spent more time in an area that did not correlate with their original tagging area. Of these, two birds had been tagged in the front of the house and spent the majority of their time in the middle area, one had been tagged in the front of the house and spent the majority of their time at the back, and one bird had been tagged in the back of the house and spent the majority of their time at the front. The preference of birds for centre or edge locations was less clear (Table 1). For the 7 birds initially tagged in edge areas, 5 of those spent the majority of their time in edge zones, while only 6 out of 10 centrally tagged birds spent the majority of their time in central areas.

To determine whether broilers spent more of their time in a smaller “territory” while still moving around a larger area, we calculated the time that broilers spent in proximity to the centre of the zone they were initially tagged in (Fig. 4e). Individual variation was also shown in this measure (Table 2). For example, two broilers remained in a small area, spending over 90% of their time within a 10 m distance from the centre of the zone they were initially tagged in. Other broilers spent their time more evenly in a large area, with one spending most of their time over 30 m away from their initial tagging zone. Overall, 8 broilers spent over 50% of their time within a 10 m distance of their tagging zone and 14 broilers spent over 50% of their time within a 20 m distance. Heat maps generated for tagged broilers also highlighted the individual variation in their use of the house (Fig. 4a,b). A heat map created for all tagged broilers during the pre-thinning period, to determine whether there were certain areas of the house that were favoured, showed no discernible pattern (Fig. 4c).

**Table 2:** The time (%) that Ross 308 broilers wearing ultra-wideband tags spent in proximity to the centre of the zone (4.26 × 4.02 m) that they were initially chosen from at the beginning of the study.

Distance from initial tagging zone	Tag number																
	3	5	7	8	9	10	12	13	14	15	16	18	19	22	23	24	25
Within 10 m	60	25	29	18	76	13	26	28	0	94	95	41	34	61	51	67	69
Within 20 m	98	78	51	49	99	35	72	83	0	99	100	77	90	86	97	83	82
Within 30 m	100	93	62	64	100	41	94	91	1	100		85	96	95	100	90	99
Within 40 m		97	63	79		48	97	100	20			89	99	97		99	100
Within 50 m		99	69	98		56	100		33			97	100	100		100	
Within 60 m		100	94	100		71			55			98					
Within 70 m			100			89			78			100					
Within 80 m						94			95								
Within 90 m						100			100								

The tagging of birds was staggered, with tags 3–13 observed for nine days before thinning from Day 22 until Day 31 and tags 14–25 observed for six days from Day 25 until Day 31.

#### Distribution of broilers after their release from thinning pen

**Figure 6:**
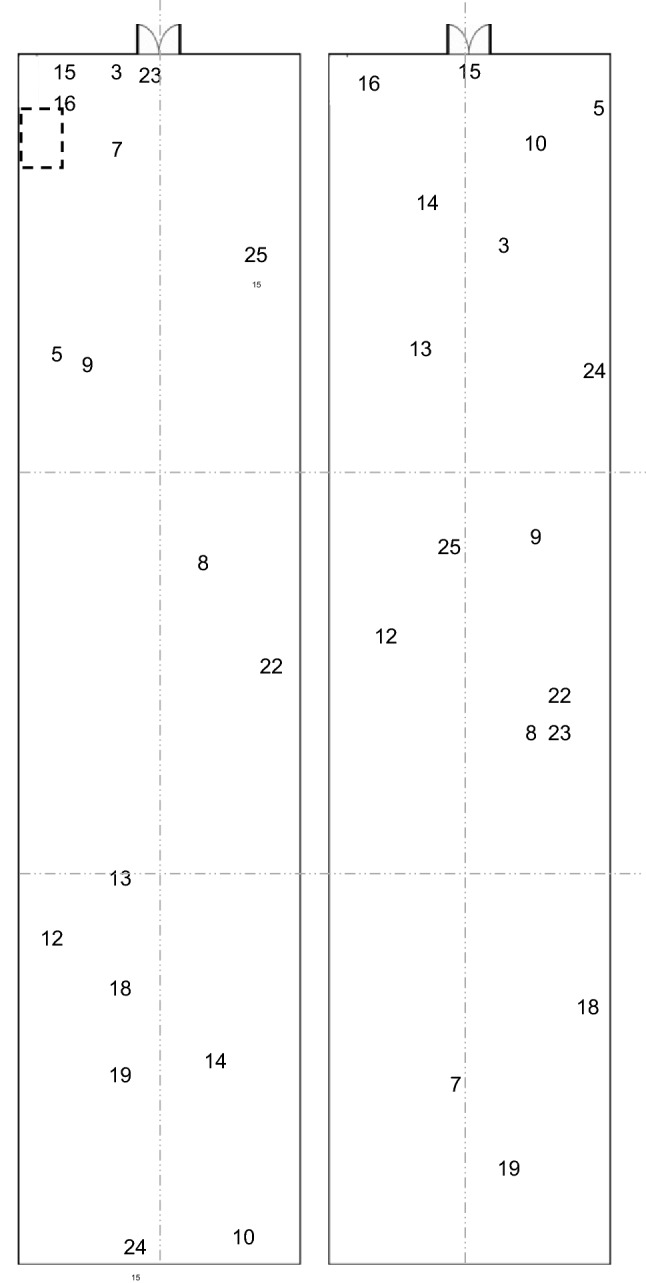
The location of all tagged broilers at 12 00 h on the day they were collected and penned for thinning on Day 31 (left) and directly before their backpacks were removed at 09 00 h on Day 38 before clearing (right). The dashed box indicates the location of the temporary pen. Dash-dot-dot lines indicate the split of the house into left/right (right is closest to the temporary pen) and front (section closest to the temporary pen), middle and back.

Broilers were collected and penned in the front of the house to prevent their removal at thinning on Day 31, then released after 21 h for a further 7 days before they were collected for clearing (Fig. 6). Of the nine broilers that were collected from the middle and back areas of the house, three tags (8, 18 and 22) returned to the main area they had been collected from (middle, back and middle, respectively) within 24 h. One further tagged bird (19) returned to the back of the house after 4 days. Tags 8, 18 and 19 then spent the majority of their time until clearing in their original area, while tag 22 had been collected from the middle of the house but spent the majority of their time at the back. The remaining five birds (tags 10, 12, 13, 14 and 24) never returned to the area they were collected from.

Of the eight broilers that were already in the front of the house when they were collected (Fig. 6), three birds left and moved into the middle of the house within 24 h. At clearing, two broilers had never left the front of the house (tags 15 and 16) while the remaining birds had travelled into the middle of the house at least once. However, five out of the eight broilers spent the majority of their time in the front of the house before clearing. As would be expected, more data points were recorded in the front of the house, where the birds had all been released, for a heat map of post-thinning movement (Fig. 4d).

### Associations between physiology and movement

The observation period before tagged broilers were penned to protect them from thinning provided the clearest overview of their natural spread around the house. During this time, there was no clear correlation between broiler start weight or end weight and the percentage of zones that they occupied (p > 0.2 for all). There was also no significant correlation between broiler start weight or end weight and the percentage of time they spent in edge zones of the house during the same period (p > 0.6 for all). The majority of tagged broilers had a final gait score of 2 (12 out of 17), with four broilers with a final gait score of 1, and one with a gait score of 3. There was no clear pattern between gait score and the percentage of zones occupied, the distance travelled or the time they spent in edge zones (Table 1). Broilers with a final gait score of 1 (N = 4) occupied between 43 and 97 zones (M = 75.3), with pre-thin recorded distances of between 6 186 m and 10 852 m (M = 8989.5 m) and time spent in edge zones ranging from 42 to 71% (M = 50.7%; Table 1). The majority, with a final gait score of 2 (N = 12), occupied between 21 and 82 zones (M = 51.4), with pre-thin recorded distances of between 5 536 m and 13 438 m (M = 10 545.6 m) and time spent in edge zones ranging from 16 to 90% (M = 56.6%; Table 1). The only broiler with a gait score of 3 was present in 69 of the house zones, recorded travelling 8 042 m during pre-thinning and spent 46% of their time in edge zones. This meant that, for example, the broiler with the highest final gait score was recorded in a higher number of zones and over a larger recorded distance than a broiler with a final gait score of 1. Overall, there was no significant correlation between broiler start weight or end weight and pre-thin distance travelled (p > 0.1 for all).

### Movement by age

**Figure 7:**
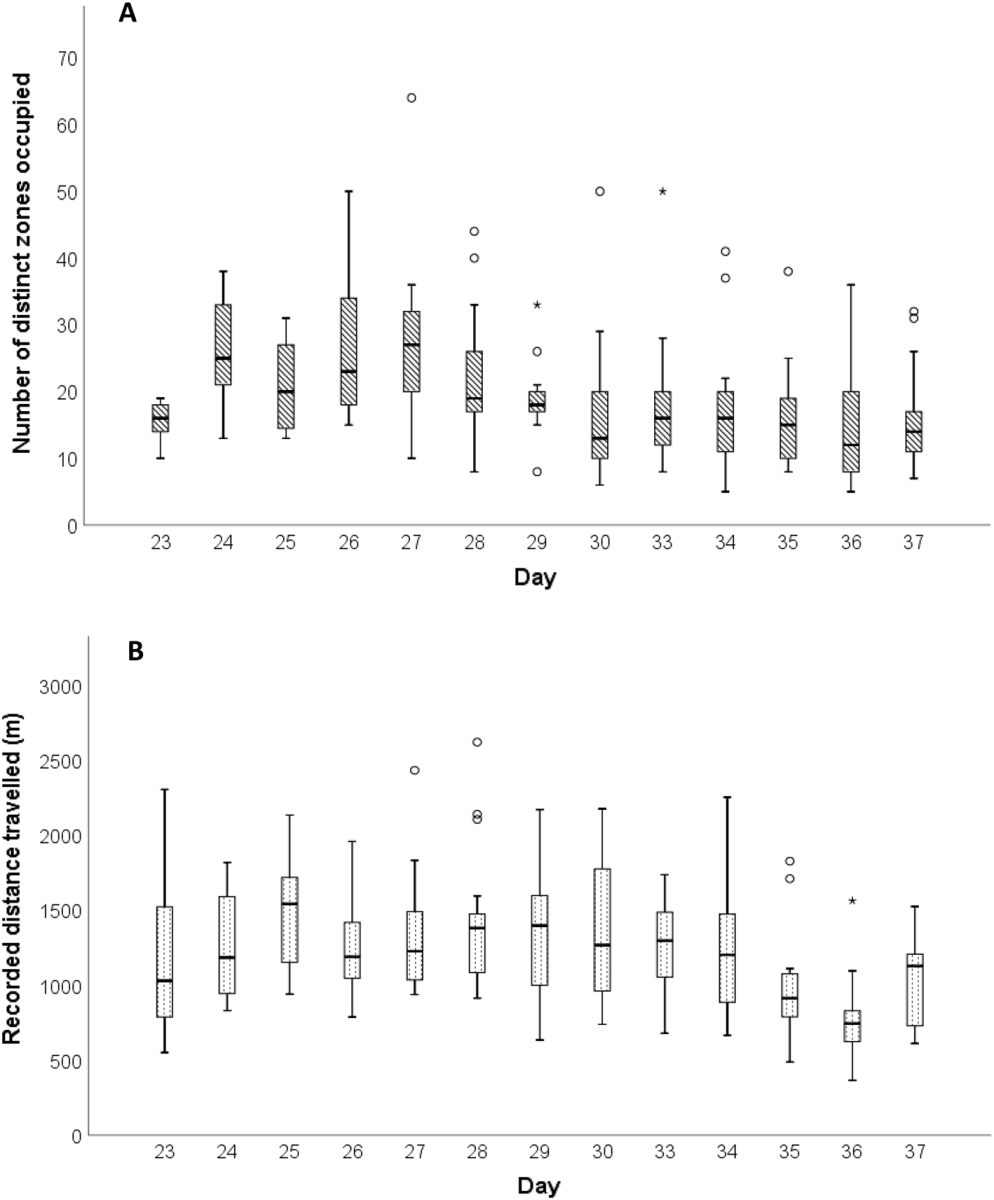
The average number of distinct zones that a broiler moved into each day (**A**) and the average recorded distance travelled by broilers across the cycle (**B**). (**A**) Represents a measure of space use, rather than activity levels, for example if they travelled repeatedly from zone 1 to zone 2 it would only be considered two distinct zones. Distances are expected to be inflated due to the accuracy of the UWB system (± 30 cm) but represent general patterns. Data for Days 31 and 32 are excluded as broilers were penned for a part of these days to prevent them from being removed during thinning. As the tagging of broilers was staggered, Days 23–25 consist of data from 8 tags while Days 26–37 consist of data from all 17 tags.

The average number of distinct zones occupied and average distance travelled per day varied across the production cycle (Fig. 7), with generally less movement towards the end of the cycle. A total of 14 out of 17 broilers were recorded in more zones in the four days before thinning compared to before slaughter, indicating a reduction in space use or “ranging” in older birds (Table 3). Similarly, 15 out of 17 broilers showed a reduction in distance travelled, indicating a reduction in activity levels, across the same periods (Table 4). However, there remained large variation between days and between individuals for both measures (Tables 3, 4). For example, approximately half of broilers showed a reduction in both recorded distance and distinct zone use between Day 27 and Day 30, while the remaining broilers either showed contrasting patterns or an increase in both.

**Table 3:** The number of distinct zones that broiler chickens occupied in an indoor commercial broiler house (85 m × 20 m).

Tag^1^	Day														
	23	24	25	26	27	28	29	30	A^†^	33	34	35	36	37	A^‡^
3	10	22	16	18	12	17	16	10	13.8	8	12	15	16	25	17.0
5	15	13	31	35	28	25	21	13	21.8	20	22	23	8	12	16.3
7	18	34	25	50	32	28	18	10	22.0	28	10	17	22	7	14.0
8	19	26	13	38	27	25	26	20	24.5	19	16	8	9	11	11.0
9	13	20	13	23	13	17	18	17	16.3	20	21	15	12	12	15.0
10	18	32	18	32	31	24	17	18	22.5	8	13	16	8	11	12.0
12	16	38	29	34	31	19	16	15	20.3	11	15	10	8	14	11.8
13	16	24	22	27	20	16	8	29	18.3	15	16	16	17	8	14.3
14				23	27	17	19	13	19.0	12	11	13	18	14	14.0
15				16	33	17	20	7	19.3	10	5	10	6	7	7.0
16				19	10	8	19	6	10.8	12	6	9	10	10	8.8
18				37	64	44	33	50	47.8	50	41	38	36	32	36.8
19				17	33	40	18	9	25.0	16	20	21	7	17	16.3
22				33	36	33	17	25	27.8	16	37	25	20	31	28.3
23				19	10	13	15	13	12.8	28	19	19	23	26	21.8
24				15	24	16	17	8	16.3	21	16	10	5	15	11.5
25				17	24	26	21	21	23.0	19	11	14	22	16	15.8

The house was virtually split into 100 small zones, 4.26 × 4.02 m, and broilers wore ultra-wideband tags to record movement across the observation period. Days 31 and 32 are excluded as broilers were penned for part of the day during thinning. Distinct zones represent the level of space use rather than activity levels, for example if they travelled repeatedly from zone 1 to zone 2 it would only be considered two distinct zones. A^†^ displays the average (mean) number of distinct zones occupied in the four days before thinning (Days 27–30), while A^‡^ displays the average number of distinct zones occupied in the four days before slaughter (Days 34–37).

^1^The tagging of birds was staggered, with data available for full days from Day 23 for tags 3–13 and Day 26 for tags 14–25.

**Table 4:** The recorded distance that Ross 308 broiler chickens wearing ultra-wideband tags travelled by day in an indoor commercial house, across the observation period.

Tag^1^	Day														
	23	24	25	26	27	28	29	30	A^†^	33	34	35	36	37	A^‡^
3	766	1262	1713	1192	1140	1598	1160	1018	1229.0	1695	667	916	696	1028	826.8
5	2310	1822	1496	1049	1398	1313	1210	964	1221.3	844	1213	703	477	1131	881.0
7	1162	866	943	952	1230	1087	1445	2181	1485.8	1407	1489	1078	747	615	982.3
8	1183	1701	2139	1404	1339	1455	965	1079	1209.5	791	880	832	771	710	798.3
9	1869	1026	1595	1706	990	1478	845	1694	1251.8	1086	771	594	574	1070	752.3
10	902	1487	1732	1061	1719	1156	642	815	1083.0	681	889	489	537	613	632.0
12	814	832	1092	1195	1145	1083	1601	1536	1341.3	1440	1007	946	801	663	854.3
13	553	1113	1217	1042	1006	1292	636	1270	1051.0	1266	1477	791	864	1528	1165.0
14				897	1280	1044	1401	900	1156.3	1057	726	882	680	1148	859.0
15				1954	1836	2144	1958	2057	1998.8	1300	1205	1113	1098	1460	1219.0
16				1963	2438	2626	1647	2089	2200.0	1638	2258	1831	1023	1438	1637.5
18				1339	1643	1419	1449	1118	1407.3	843	1213	979	831	1210	1058.3
19				790	940	916	1028	741	906.3	1490	972	833	366	731	725.5
22				1422	1495	2110	2175	1864	1911.0	1726	1095	594	677	1195	890.3
23				1586	1000	1407	1954	1300	1415.3	1271	1644	1714	801	1201	1340.0
24				1139	1038	975	1412	915	1085.0	1358	1385	928	1567	1210	1272.5
25				1151	1216	1384	1001	1780	1345.3	1740	1957	1105	626	931	1154.8

Days 31 and 32 are excluded as broilers were penned for part of the day during thinning. A^†^ displays the average (mean) recorded distance travelled in the four days before thinning (Days 27–30), while A^‡^ displays the average recorded distance travelled in the four days before slaughter (Days 34–37).

This is the distance recorded by the ultra-wideband system which has an accuracy of ~ 30 cm. As such, distances may be inflated and are used to represent patterns of movement rather than exact distances travelled.

^1^The tagging of birds was staggered, with data available for full days from Day 23 for tags 3–13 and Day 26 for tags 14–25.

## Discussion

The purpose of this study was to remotely track the movement of individual broiler chickens for the final two weeks of the production cycle. We successfully recorded data from 17 tagged birds using the RTLS and ultra-wideband backpacks. Contrary to our expectations that the homogenous breed characteristics and housing system would lead to only minor differences between the birds, there was significant individual variation in the movement patterns of individual broilers that was not clearly explained by variations in their physiology.

One of the main aims of this study was to determine how much of a large commercial house broilers use and to advance the existing debate on whether intensively reared poultry create “home ranges” or “site attachments” within commercial housing. Overall, we found significant individual variation in broiler movement patterns, with some broilers exploring the majority of the house and others restricting themselves to small areas. In general, broilers were detected in a large area of the house over a relatively short period of the production cycle (6 or 9 day observation period before thinning). The majority of broilers were detected in over half of the house during this time, with two broilers visiting over 90% of the 1700 m^2^ house. Individual variation was also apparent in our investigation of location preferences. Although we found that most broilers spent the majority of their time in the same main area (~ 560 m^2^; front, middle or back of the house) that they were initially tagged in, two broilers spent over 90% of the observation period within 10 m of the centre of the zone they were initially found in, while another was recorded far from this initial zone for the entire period. A general preference for spending time towards the edge of the house was noted among tagged broilers. Overall, broilers spent more of their time in edge areas (55%) compared to central areas (45%), despite there being more central sections in the house. Birds initially tagged in edge areas were also more likely to spend the majority of their time at the edge of the house compared to those initially tagged in central areas, suggesting that particular broilers maintained a stronger edge preference than others. However, there was also wide variation between individual broilers, with two birds spending > 85% of their time and one < 16% of their time in edge locations. Several studies have found that some broilers will group closer to the pen walls than expected by chance^3,15^, with suggestions that this may be to facilitate resting^15,16^ or as an anti-predator behaviour^17^. The physiological measures taken in this study were not able to explain the individual variation present, with no clear link between the amount of time broilers spent in edge locations and their gait score or body weight.

An assumption of this study is that the initial tagging location for broilers at the start represented their “preferred location”. As we were only able to fit backpacks on broilers that were around 21 days of age, we do not have historical information about their movements before this time. Also, as broilers were tagged one after the other, there were some broilers that were tagged after the observers had been in the house for some time, increasing the risk that they would have been displaced from preferred areas. However, there remained a clear individual variation in the movement patterns and space use across the observation period, which does not support suggestions that broilers consistently create smaller territories within their flocks. For example, McBride and Foenander^6^ found that chickens in a flock of 80 birds reared in a 6 × 11 m (66 m^2^) house used about a third of the available space, created clear territories and only briefly strayed across boundaries. Further evidence for poultry creating some form of “site attachment” came from studies on laying hens^7,8^ and breeder flocks^9^. Craig and Guhl^8^ reported that a flock of 400 pullets showed preferences for particular areas of their house, while Crawford^7^ found that laying hens tended to return to the area of the pen they had initially been brooded in. Pamment et al.^9^ similarly reported site preferences for cockerels in breeding pens, with more dominant males generally moving over smaller areas than low-ranking males in larger pens. However, Appleby et al.^2^ argued that these studies do not provide conclusive evidence that poultry create territories in larger flocks, pointing out the ill-defined home ranges and small flock sizes described in previous research. In their own study of broiler breeders with numbered leg tags, they found that individual birds among flocks of 4000 typically used more than half the available space (around 505 m^2^) and neither sex appeared to restrict themselves to small areas of the house. We similarly found that the majority of broilers used more than half of the available space, and that broilers tagged for longer (nine days compared to six days before thin) were recorded in more of the house, suggesting their true use of the space is likely to be larger across the entire production cycle.

There was further opportunity to assess the strength of location preferences after thinning by determining whether broilers would return to the area they had been collected from once released from a pen at the front of the house. As with other measures, the response of broilers to release was varied and it is difficult to determine whether their movements were random or represented individual variation in preference stability. While three out of nine broilers collected from the middle or back of the house returned to their proximate original locations within 24 h, five never returned to their original location. Of those who were already at the front of the house when collected, five out of eight then generally remained in the front of the house once released. A larger sample size of tagged broilers would be needed to explore space use and location preferences further, however the wide variation in movement patterns suggests there are additional factors influencing broiler roaming. Despite flocks of broilers appearing to be generally homogenous, individual traits are also likely to influence broiler movement patterns outside of their physiology. While some broilers in free range housing show more willingness to forage and to visit outdoor ranges, others show lower motivation to contrafreeload and will consistently remain inside^18,19^. Broilers also vary in their cognitive flexibility and their ability to complete spatial and non-spatial memory tasks^20–22^. Consistent with other species, it is likely that broilers would demonstrate a diverse set of personality and cognitive traits that could be linked to movement patterns^23–25^. For example, less fearful mallards spent more time exploring a maze before reaching the food reward compartment^26^, starlings that fed faster in a novel environment were also faster at solving a learning task^27^, and an age-dependant association between exploratory behaviour and learning speed was found in red jungle fowl^28^. Although gender is also a possible source of variation, broilers are slaughtered before sexual dimorphism is clear and sex has not been found to be predictive of roaming behaviour in broilers^18^. Additional personality tests for individually tracked broilers would be valuable in unpicking the causal factors for their individual variation in movement patterns.

It is widely accepted that broiler activity levels decrease with age and that worse gait scores and higher body weights are associated with lower levels of activity^10,11^. Gait scores of 3 and above are thought to be particularly limiting due to the possible pain associated with this level of abnormality^29,30^. The conformation of modern broilers has also made any movement for them energetically costly which, in addition to low levels of environmental stimulation and the proximity of resources, has altered their movement patterns and made them highly sedentary compared to their ancestors and other poultry^31–33^. However, the level of movement for some broilers in this study was surprisingly high and, contrary to our expectations, we found no clear pattern between space use or activity levels and gait score or final weight. Although all broilers initially chosen for tagging had either a gait score of 0 or 1, they varied in starting weight between 875 and 1410 g, which we would have expected to have a clearer effect on their general movement. There was a general reduction in activity levels and space use with age as expected, but this pattern was not linear and there remained significant variation between days and between broilers. In fact, there was not even a clear association between space use and activity levels, meaning that broilers that occupied a larger area of the house were not necessarily more active than those that were only recorded in a small area of the house.

The only broiler displaying lameness (gait score 3; GS3) did not record the lowest space use or activity levels across the cycle compared to broilers with a better walking ability. It is likely that because this poor gait was only displayed at the end of the production cycle, the broiler was only affected in the final few days rather than representing a worse gait score across the cycle. They did record a reduction in distance travelled in the final few days which appears to confirm this, however there was no associated reduction in space use, suggesting they still occupied a similar sized area of the house. A larger sample of tagged broilers would be needed to explore this further, however it is possible that continuous recording of data has revealed a more complicated relationship between activity and welfare measures compared to standard interval observations. Aydin et al.^34^ found a similar lack of clear linear relationship between gait score and activity in broilers kept in small (1 m × 1 m) pens. Using an automated image monitoring tool across five days, they found no difference in the activity levels of birds with a gait score of GS0-GS2 and that heavier broilers with GS3 actually had the highest levels of activity before dropping off for GS4 and GS5^34^. Using similar UWB technology to the present study, van der Sluis et al.^35^ tracked the distance commercial broilers moved in metres across 17 days in pens < 5 m^2^. Although they found general differences between those with “good gait” (GS0-2) and “suboptimal gait” (GS3 +) in terms of activity levels and body weight, they concluded that it would be difficult to predict gait scores of broilers based on their activity levels, as individual broilers within gait classifications showed differing activity levels and there was a large overlap between gait classifications.

To the best of our knowledge, this study presents the first data on the individual movement patterns of free-moving broilers reared in commercial housing. Overall, there was significant individual variation in the way tagged broilers used the available space that was not clearly explained by gait score or body weight. This wearable technology presents a significant opportunity for advancement in our understanding of the causal factors of individual variation and the complex impact of broiler physiology, personality and environment on welfare measures.

## Data Availability

The datasets generated and analysed during the current study are available from the corresponding author on reasonable request.
